# Physical Activity Patterns and Factors Related to Exercise during Pregnancy: A Cross Sectional Study

**DOI:** 10.1371/journal.pone.0128953

**Published:** 2015-06-17

**Authors:** Simony Lira Nascimento, Fernanda Garanhani Surita, Ana Carolina Godoy, Karina Tamy Kasawara, Sirlei Siani Morais

**Affiliations:** 1 Department of Obstetrics and Gynecology, Faculty Medical Sciences, University of Campinas (UNICAMP), Campinas—SP, Brazil; 2 Department of Physiotherapy, Federal University of Ceara, Fortaleza—CE, Brazil; Queen's University, CANADA

## Abstract

**Objective:**

To assess the physical activity levels of pregnant women and to examine the characteristics associated with the practice of exercise and the activities of daily living during pregnancy.

**Methods:**

For this cross-sectional study, 1,279 women were recruited within 72 hours postpartum. They were interviewed about their socio-demographic data and obstetric history and were administered self-report questionnaires about exercise and daily physical activities during pregnancy. Data on the current pregnancy, labor, delivery, and newborn outcomes were collected from participants’ medical records. To analyze factors related to the practice of exercise, we used the student t-test, X², and odds ratio (OR), with a corresponding 95% confident interval (CI), followed by a multiple logistic regression. The significance level was 5%.

**Results:**

Compared to the pre-pregnancy period, the prevalence of physical activity among participants was lower throughout pregnancy (20.1%) (p = 0.01). Half of the women interrupted practicing physical exercise due to pregnancy. The lowest prevalence of exercise was observed in the first (13.6%) and third trimesters (13.4%). Less than half of women received exercise guidance during prenatal care meetings (47.4%). Walking was the most commonly reported exercise, followed by water aerobics. Factors positively associated with exercise practice were higher educational level (OR= 1.82; CI 95% 1.28–2.60), primiparity (OR=1.49; CI 95% 1.07–2.07), exercising before pregnancy (OR= 6.45; CI 95% 4.64–8.96), and exercise guidance during prenatal care (OR=2.54; CI 95% 1.80–3.57). Mildly intense exercise and domestic activities were most frequently reported among pregnant women. There were no differences in maternal and perinatal outcomes between active and sedentary pregnant women.

**Conclusion:**

The findings indicate that promoting physical activity remains a priority in public health policy, and women of childbearing age, especially those planning a pregnancy, should be encouraged to adopt an exercise routine or maintain an active lifestyle during pregnancy in order to avoid sedentary- and obesity-associated risks.

## Introduction

It is well established in the literature that an active lifestyle produces health benefits, especially for the prevention of chronic degenerative diseases [1]. For most pregnant women, exercise is not only safe for fetal health but is also associated with numerous maternal health benefits, including the prevention and control of gestational diabetes, control of excessive weight gain, reduction of lower back pain complaints, and positive effects on maternal mental health and quality of life [2–5]. Therefore, regular exercise is recommended for all healthy pregnant women. Those who were active before pregnancy and those who were sedentary but want to begin some activity during pregnancy can practice physical activity, provided that they engage in activities of moderate intensity and avoid those that present a risk of falling or abdominal trauma [6,7].

The consensus that physical activity is not harmful to pregnancy and is important for health and wellness has led some women to choose to stay active during pregnancy. However, the literature indicates that most pregnant women do not practice any form of exercise and tend to decrease their level of physical activity, including household and occupational activities [8–11]. Pregnant women have cited discomfort during exercise, fear of harm to the fetus, and a history of abortion or infertility treatments as reasons for reducing physical activity [12–14]. Among socio-demographic factors, lower educational level and income, greater number of children at home are most frequently associated with reduced physical activity [15].

Studies in different populations have produced mixed results, depending on the method used to assess the prevalence and intensity of exercise [16–19]. Among pregnant Brazilian women, earlier studies found high physical inactivity rates during pregnancy, leading to a completely sedentary lifestyle in the third trimester of pregnancy [8–10].

Brazil is a huge country, with large regional differences, social inequalities, and socio-economic and demographic changes. Although life expectancy has grown longer, it has been accompanied by increased rates of chronic diseases and obesity, including among reproductive-age women, which is majority of the population of women. Considering the priority to increase physical activity levels in the general population, it is necessary to study the actual status of physical activity in different groups and the factors related to its practice. In the case of pregnant women, interest is increased by this especially important moment in a woman’s life, which can have effects on her health in the future.

Mostly in population-based studies, there is a correlation between the use of objective measurement instruments, such as accelerometers and pedometers, and the application of questionnaires to assess the level of physical activity [20–23].

This study was aimed at assessing the prevalence of physical activity among pregnant women in the city of Campinas in southeastern Brazil and to verify what characteristics are related to exercise during pregnancy. Additionally, maternal and perinatal outcomes were evaluated.

## Material and Methods

A cross-sectional study was carried out in the city of Campinas city in the state of São Paulo in southeastern of Brazil. Data collection was performed from October 2011 to February 2014.

### Subject Selection

Potential participants were approached between 12 and 72 hours postpartum. Eligibility criteria were postpartum women who lived in Campinas and had a hospital birth, single pregnancy, and live newborn. Women who presented difficulty with written or verbal comprehension or had physical or psychological conditions that could interfere with comprehension and/or autonomy in consent to participate were excluded.

Eligible participants were identified through a standardized chart review. They were invited to participate in this study and signed a consent form confirming their agreement to participate. This study protocol received approval from the University of Campinas Ethical Committee under registration number 991/2011.

### Target Population

Campinas is the third major city in São Paulo, with approximately 1.5 million inhabitants and a mean of 15,000 deliveries per year. The sample size was based on the number of deliveries per year (single pregnancy) among Campinas residents, estimated at 14,693 in 2010 [24].For the sample size, the highest variability possible based on annual deliveries was calculated, resulting in p = 50% (0.5), level of significance of 5%, sampling error of 3%, and, finally, n = 995. The sample was divided among the three major maternity wards which cover 85% of births in the city, according to the proportions of annual deliveries. Those maternities were funded by the public health care system, Supplementary Health System, or both. We excluded women who had home births, since in Southern region of Brazil it corresponds to just 0.22% of total births [24]. A sample larger than the minimum necessary was recruited to protect against possible data loss.

### Data collection

Data collection was performed at each maternity ward on pre-specified days in order to ensure the required sample size. At the day of data collection in established center, all eligible and available women were invited to participate in the study. Women were interviewed during clinic visits using standardized questionnaires: a questionnaire on their socioeconomic status, anthropometric data, and obstetric history; a questionnaire on physical exercise during pregnancy; and a questionnaire on physical activities, including daily physical activity, specifically for pregnant women. Data on participants’ pregnancy period, co-morbidities, delivery, and newborn outcomes were collected from medical records and prenatal care cards.

A questionnaire specifically addressing physical exercise practice was developed for this study and included questions about exercise practice in each gestational trimester, including type, duration, frequency, and intensity of exercise (adapted Borg scale) [25]. In addition, questions on guidance on exercise practice received during prenatal care meetings was administered [S1 File]. Physical exercise was defined as structured, planned, repetitive physical activity intended to promote health and maintain one or more components of physical ability [26].

This study also used the Pregnant Physical Activity Questionnaire (PPAQ) validated for the Portuguese language in Brazil [9]. The type, intensity, duration, and frequency of physical activity were recorded as minutes and hours per day. Each activity was classified according to intensity in Metabolic Equivalent Task (MET)—sedentary (<1.5 METs), low (1.5 a <3.0 METs), moderate (3.0 a 6.0 METs), and vigorous intensity (>6.0 METs)—and type—labor, domestic (e.g., caring for a person, home care), and sports/exercise [20]. Participants were instructed to answer this questionnaire based on activities performed in the last three months, or their third trimester of pregnancy. Physical activity was considered to be any voluntary, corporal movement that increased the metabolism above its resting rate [27].

Quality control was performed monthly at each center and during site visits by the coordinating center team.

### Statistical analysis

The results are described with the mean and standard deviations (SD) for continuous outcomes with normal distribution; the median and variation for continuous outcomes with non-normal distribution; and the frequency and percentage for categorical outcomes. All pregnant women were classified as either active, those who reported performing exercise regularly (twice or more per week, at least 30 minutes per session) at least in one trimester, or sedentary, those who did not exercise at all or do not achieve active criteria. All analyses ware based our own exercise questionnaires. PPAQ was used just for descriptive propose.

All bivariate and multivariate analyses were done based on these two categories. Further, active women were classified according a cut-off point of at least 150 minutes of exercise per week in accordance with ACOG guideline [6], but as very few women achieve this criterion, it was used just for descriptive propose. McNemar’s Chi square test was used to analyze the difference between levels of physical exercise during pregnancy (before pregnancy versus during pregnancy and versus the three trimesters). The association between the variables and the practice of physical exercise was tested using a student’s t-test (for continuous variables) and chi-square test (for categorical variables). To estimate the chance of physical exercise, the odds ratio (OR) and corresponding confidence interval (CI) of 95% were calculated. Then a multiple logistic regression, with selection criteria of stepwise variables, was performed. The level of significance was assumed to be 5%. The software used to perform statistical analysis was Epi-info version 5.1 and SAS version 9.2.

## Results

Between October 2011 and February 2014, 1791 women were assessed for eligibility, 476 did not fulfill inclusion criteria, then 1315 were invited to participate in the study, 36 (2.7%) declined participation, thus 1,279 women were included in this study. Participants’ demographic characteristics are described in Table 1. Regarding the practice of physical exercise before pregnancy, 23.2% of women reported some type of exercise during this period. However, 55.2% of these women stopped exercising due to pregnancy, while 29.3% maintained their practice during pregnancy, and 15.5% kept exercising practice but decreased the intensity and frequency.

**Table 1:** Demographic and obstetric characteristics of women.

Variables	n = 1279
**Age**—mean ± SD	27.13 ± 6.37
<19 years—n (%)	166 (12.98)
20–34 years—n (%)	929 (72.63)
35–39 years—n (%)	149 (11.65)
>40 years—n (%)	35 (2.74)
**Color/ethnicity**—n (%) ^a^	
White	606 (47.5)
Non-white	670 (52.5)
**Educational level**—n (%) ^a^	
Elementary school	271 (21.3)
High School	725(57.0)
College, University, or Advanced Degree	277(21.7)
**Worked During Pregnancy**—n (%)	691(54.15)
**Lives with a partner**—n (%)	1198 (93.67)
**Planned Pregnancy**—n (%)	638(49.96)
**Parity**—n (%)	
1	608 (47.54)
≥ 2	671(52.46)
**Gestational age at 1^st^ Prenatal visit**—mean ± SD	12.16± 6.21
**Prenatal visits**—mean ± SD	8.79±2.42
**Public Prenatal care**—n (%)	885 (69.63)
**Smoking During Pregnancy**—n (%)	102 (8.0)
**Diabetes**—n (%)	78 (6.14)
**Maternal hypertension**—n (%)	118 (9.26)
**Pre-gestational Weight** (Kg)—mean ± SD	64.62 ± 14.16
**Pre-gestational BMI** (kg/m2)—mean ± SD ^a^	24.62 ± 5.06
< 18.5—n (%)	82 (6.5)
≥ 18.5 < 25—n (%)	689 (55.2)
≥ 25 < 30—n (%)	308(24.6)
≥ 30—n (%)	169 (13.5)

^a^-Cell counts may not add up to total number of subjects due to missing values.

**Fig 1:**
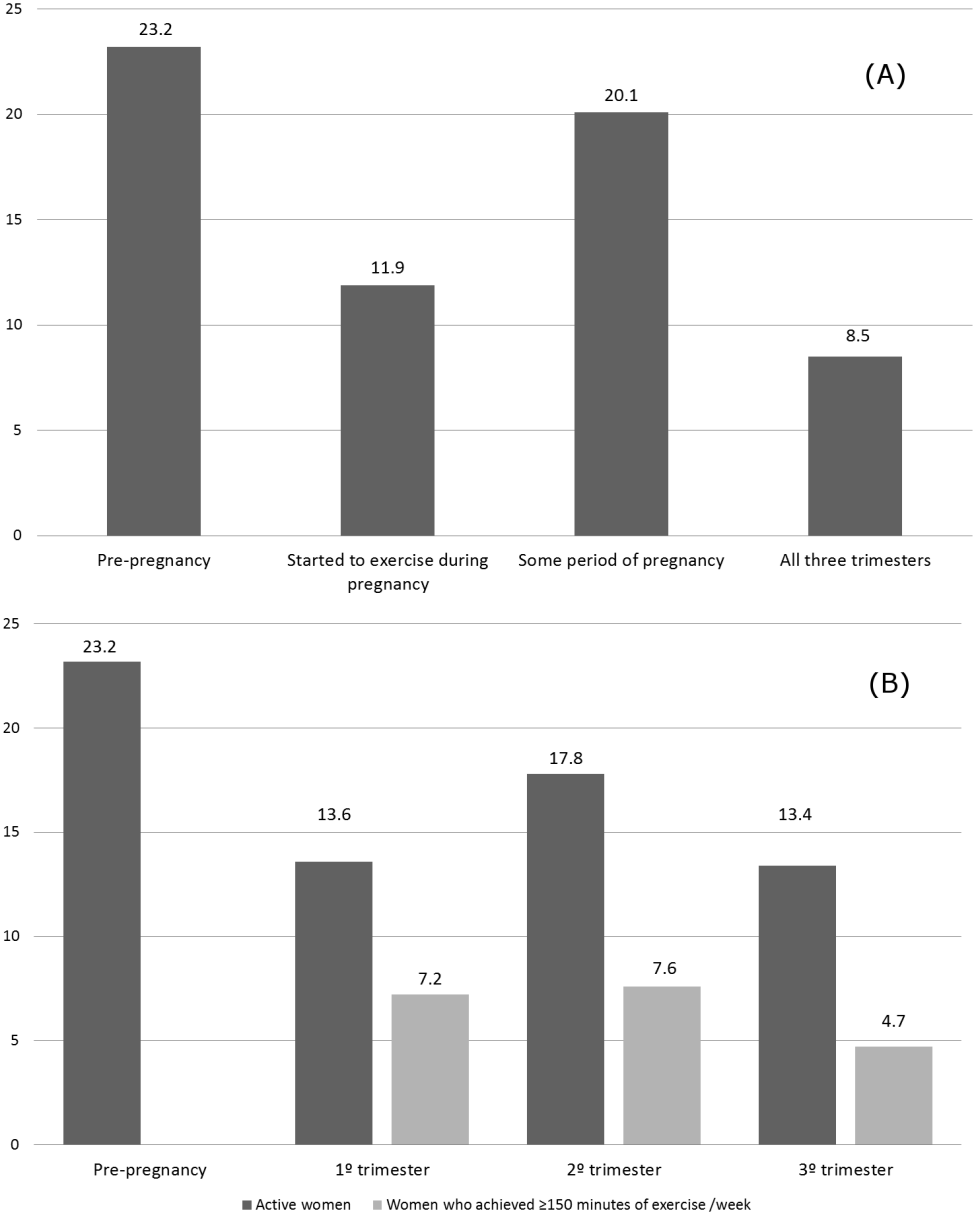
Prevalence of exercise practice before and during pregnancy in different gestational period and according to exercise criteria: active or ACOG criteria. (A) Frequencies of pre-gestational exercise vs. exercise some period of pregnancy (p = 0.01); pre-gestational exercise vs. all three trimesters (p<0.0001). (B) Pre-gestational exercise vs. exercise at 1^st^, 2^sd^ e 3^tr^ trimesters (p<0.0001); exercise 1° vs. 2° and 2° vs. 3° trimesters (p<0.0001)—McNemar Qui-square test. Frequency of exercise according to ACOG recommendations (150 minutes/week).

The prevalence of exercise practice was lower during than before pregnancy (p = 0.01): 20.1% women reported practicing exercise during some period of pregnancy. Considering that the prevalence was lower in the first trimester (13.6%) and the third trimester (13.4%) and higher in the second trimester (17.8%) (p<0.0001), only (8.4%) women remained active throughout all three trimesters of pregnancy (Fig 1). The proportion of women who completed the minimum of 150 minutes of aerobic exercise per week was even lower: 7.2%, 7.6%, and 4.7% in the first, second, and third trimesters, respectively (Fig 1).

Less than half of participants (47.4%) received physical exercise guidance during prenatal visits, and 14.9% were told to stop exercise. The most frequent guidance method was individualized conversations with a physician during prenatal care (95.2%). Approaches involving groups, leaflets, video, and other health professionals (e.g., physical therapists, physical educators, nurses) were minimal (data not shown in a table). From 981 women who were sedentary before pregnancy, 117 (11.9%) started exercising during pregnancy.

**Fig 2:**
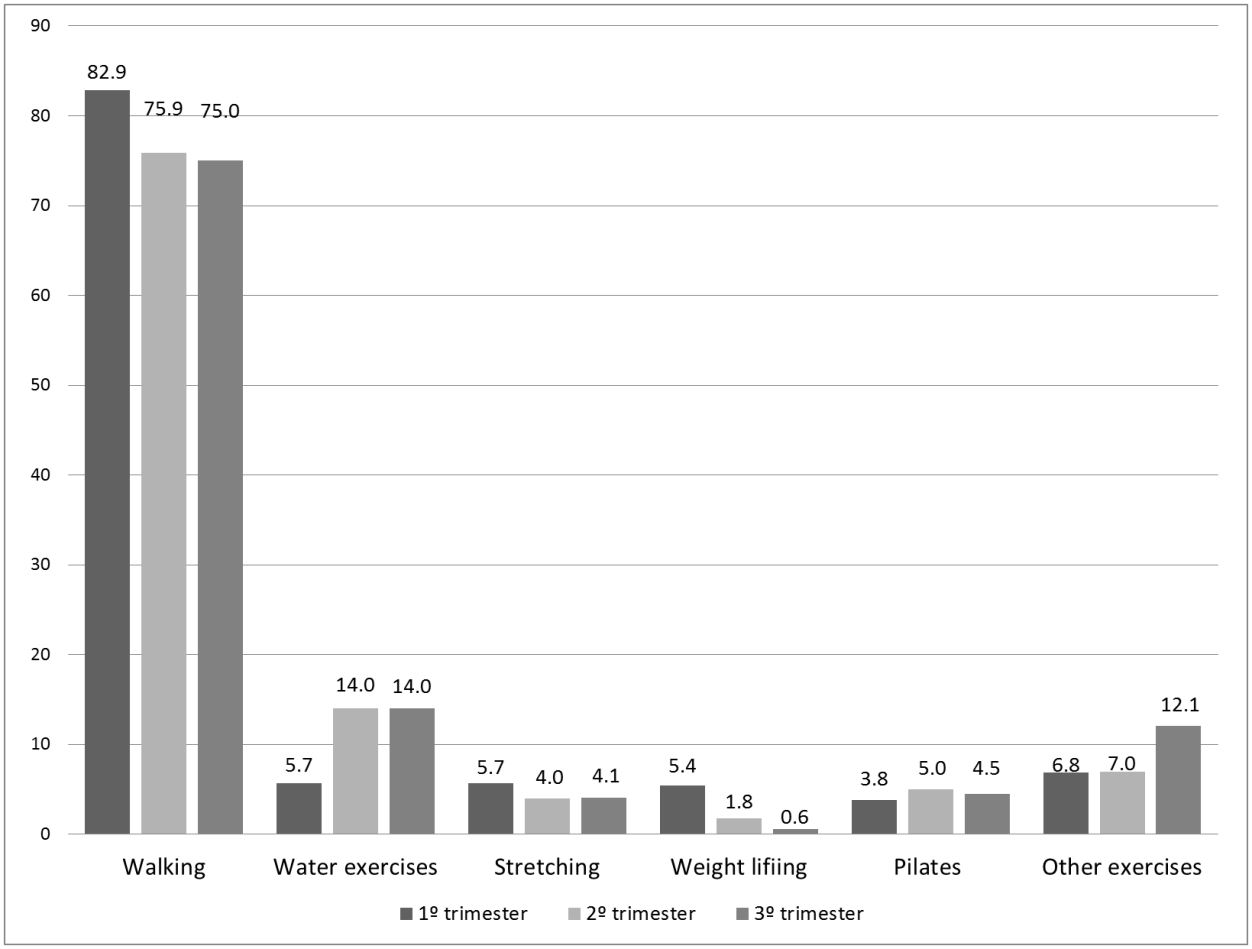
The percentage participating in a given exercise type in each gestational trimester. Other exercises: dance, cycling, swimming, running, prenatal exercise class e pelvic floor exercises.

The most common activity reported during all three trimesters was walking, with the greatest frequency during the first trimester (82.2%). The second most common activity was water aerobics, with the greatest frequency during the second and third trimesters. Other types of exercise reported were stretching, Pilates, yoga, dance, weight lifting, biking, swimming, aerobics prenatal class, and pelvic floor exercises (Fig 2). Average frequency in days/week of all types of exercise compiled was 4.23±2.0, 3.77±1.9 and 3.71±1.88 and mean duration was 51.27±36.70, 48.73±28.94, and 46.00±26.20, respectively in the 1°, 2° and 3° trimesters.

The analysis of PPAQ data on the intensity of physical activity in the third trimester shows that pregnant women expended the most energy in activities considered mild, which were the most commonly performed. Vigorous activities were mentioned rarely. Regarding type, domestic activities accounted for the largest and sports/exercise the lowest share of activity during pregnancy (Table 2).

**Table 2:** Description of physical activity energy expenditure in MET-h/week during pregnancy, according to intensity and type of activity based on PPAQ.

Physical activities (MET-h/week)	Mean	SD	Median	IQ25%	IQ75%
**Intensity**					
Sedentary	43.2	25.7	41.5	21.0	58.8
Mild	89.3	63.9	77.5	43.8	127.9
Moderate	40.0	59.6	7.0	0.0	63.0
Vigorous	0.9	6.7	0.0	0.0	0.0
**Type of Activity**					
Sports/exercise	4.2	15.4	0.0	0.0	0.0
Occupational	31.5	44.4	0.0	0.0	49.0
Household/caregiving	84.3	100.8	44.5	4.0	122.9

MET = metabolic equivalent task; PPAQ = Pregnancy Physical Activity Questionnaire.

The factors related to exercise during pregnancy were investigated by comparing the characteristics of women who exercised at some time during pregnancy (n = 258) and those who did not perform any exercise (n = 1021). It was observed that higher or graduate education, paid work, planned pregnancy, primiparity, prenatal care in the private service, exercising before pregnancy, and guidance on prenatal exercise significantly increased the odds of exercising during pregnancy (Table 3).

**Table 3:** Women characteristics according to physical activity during pregnancy (active or sedentary).

VARIABLES	ACTIVE	SEDENTARY	p	OR (CI 95%)
	N = 258	N = 1021		
**Age**—mean ± SD	27.15 ± 6.11	27.12 ± 6.43	0.9607	-
**Pre-gestational Weight**—mean ± SD	65.27 ± 14.84	64.45 ± 13.99	0.4052	-
**Pre-gestational BMI**—mean ± SD	24.45 ± 5.12	24.66 ± 5.04	0.5517	-
**Color/ ethnicity**—n (%)			0.1233	
White	130 (50.6)	476 (46.7)		1.1 (0.8–1.5)
Non-white	127 (49.4)	543 (53.3)		(ref)
**Educational level**—n (%)			<0.0001	
College, University, or Advanced Degree	99 (38.8)	178 (17.5)		3.0 (2.0–4.5)
High School	114 (44.7)	611 (60.0)		1.0 (0.6–1.5)
Elementary school	42 (16.5)	229 (22.5)		(ref)
**Working during pregnancy**—n (%)			0.0069	
Yes	159 (61.6)	532 (52.3)		1.4 (1.1–1.9)
No	99 (38.4)	486 (47.7)		(ref)
**Live with a partner**—n (%)			0.3393	
Yes	245 (95.0)	953 (93.3)		1.3 (0.7–2.4)
No	13 (5.0)	68 (6.7)		(ref)
**Planned pregnancy**—n (%)			0.024	
Yes	145 (56.2)	493 (48.4)		1.3 (1.0–1.8)
No	113 (43.8)	526 (51.6)		(ref)
**Parity**—n (%)			<0.0001	
1	157 (60.9)	451 (44.2)		1.9 (1.4–2.5)
≥ 2	101 (39.2)	570 (55.8)		(ref)
**Prenatal care type**—n (%)			0.0002	
Private	102 (39.7)	284 (28.0)		1.7 (1.2–2.2)
Public	155 (60.3)	730 (72.0)		(ref)
**Smoking pregnancy**—n (%)			0.3637	
Yes	17 (6.6)	85 (8.3)		0.7 (0.4–1.3)
No	240 (93.4)	935 (91.7)		(ref)
**Diabetes**—n (%)			0.7345	
Yes	17 (6.6)	61 (6.1)		1.1 (0.6–1.9)
No	241 (93.4)	952 (93.9)		(ref)
**Hypertension**—n (%)	92(8.8)		0.6483	
Yes	22 (8.5)	96 (90.6)		0.8 (0.5–1.4)
No	236 (91.5)	1016 (9.5)		(ref)
**Obesity**—n (%)			0,9572	
Yes	34 (13.4)	135 (13.5)		0.9 (0.6–1.4)
No	219 (86.5)	860 (86.4)		
**Exercise before pregnancy**—n (%)			<0.0001	
Yes	144 (56.0)	153 (15.0)		7.2 (5.3–9.7)
No	113 (44.0)	868 (85.0)		(ref)
**Guidance to exercise during prenatal care**—n (%)			<0.0001	
Yes	177 (69.4)	427 (41.9)		3.1 (2.3–4.2)
No	78 (30.6)	591 (58.1)		(ref)

The multiple logistic analysis showed significant associations between physical exercise during pregnancy and educational level (undergraduate or graduate), primiparity, exercise training before pregnancy, and guidance on exercise during prenatal care, all of which increased the odds of physical exercise during pregnancy (Table 4).

**Table 4:** Logistic regression results for odds of exercising during pregnancy according to women characteristics.

Variables	OR	CI 95%		p*
**Higher educational level**	1.82	1.28	2.60	0.0009
**Primiparity**	1.49	1.07	2.07	0.0197
**Exercise before pregnancy**	6.45	4.64	8.96	<.0001
**Guidance to exercise during prenatal care**	2.54	1.80	3.57	<.0001

* Independent conditions associated to exercise practice during pregnancy in the final logistic regression model: Educational level (College, University, or Advanced Degree vs. Elementary or High school); Parity (1 vs. ≥ 2); Exercise before pregnancy (yes vs. no); Guidance to exercise during prenatal care (yes vs. no).

Regarding maternal and perinatal outcomes, the average weight gain in the sample was 13.08 ± 6.08 kg, with no difference between active and sedentary women. The overall cesarean section rate was 57.4% and reached 90.8% in a maternity ward which treated only patients with health insurance; however, no association with exercise was found. Gestational age at birth and newborn weight were similar among active and sedentary women (Table 5). The newborn hospitalization rate in the neonatal intensive care unit was 3.9%. Of newborns, 95% presented an Apgar index ≥ 7 in the first minute, and only one neonatal infant had an Apgar score <7 in the fifth minute (born to a sedentary pregnant woman who had an elective C-section at 37 weeks gestation) (data not shown in a table).

**Table 5:** Perinatal outcome according to physical activity during pregnancy (active or sedentary).

Perinatal Outcome	Total sample	Active	Sedentary	
	n = 1052	n = 225	n = 827	p-value
**Gestational weight gain**(kg)—mean ± SD	13.08 ± 6.08	12.5 (5.58)	13.0 (6.9)	0.4276
**Cesarean rate**—n (%)	619(58.9)	137 (60.9)	481 (58.3)	0.2674
**GA at delivery**—mean ± SD	38.10 ± 1.38	38.72 (1.38)	38.72 (1.38)	0.9629
**Prematurity** <37 weeks—n (%)	58(5.9)	13 (5.9)	43 (5.3)	0.3674
**Newborn weight**—grams—mean ± SD	3298.12± 454.9	3244.15±454.3	3238.33±455.6	0.8671
**Newborn weight**—grams—n (%)				0.8529
<2500	59(5.8)	12 (5.5)	47 (5.9)	
2500–3999	923(90.7)	197 (90.4)	725 (90.7)	
≥ 4000	36(3.5)	9 (4.1)	27 (3.4)	

## Discussion

The findings of this study show that the prevalence of physical activity decreased with the condition of pregnancy. Half of the women stopped exercising due to pregnancy, with an even lower prevalence at the beginning and the end of pregnancy. Among women who exercised, walking was the most common type of activity. Factors associated with exercise during pregnancy included high educational level, primiparity, physical activity before pregnancy, and guidance on exercise during prenatal visits. Physical exercise did not affect maternal and perinatal outcomes.

Despite varying study designs and questionnaires, research conducted in different countries mostly agrees that women tend to reduce the level, duration, and intensity of exercise during pregnancy [11,18]. Published in 1996, one of the first studies on the prevalence of exercise among pregnant women was conducted in the United States and found that 42% of pregnant women reported practicing some form of exercise during pregnancy [16], with walking (43%), swimming (12%), and aerobics (12%) the most frequent. In a large population-based study, Evenson et al. collected data from 1,979 pregnant and 44,657 non-pregnant women and noted that 65.6% of pregnant participants reported some physical activity in the last month, compared to 73.2% of non-pregnant women [28]. However, based on the recommended 150 minutes of moderate exercise per week, the observed prevalence was 15.8% vs. 26.1%, respectively [28]. In an Irish cohort of healthy pregnant women, 21.5% followed the American College of Obstetricians and Gynecologists (ACOG) recommendations [29]. In a British population, the prevalence of pregnant women who engaged in exercise of moderate intensity for ≥3 hours per week was higher, approximately 48% in the 18th and 32nd weeks of pregnancy [17].

The findings of the present study are consistent with those of earlier studies conducted in Brazil, which also observed that few women exercise during pregnancy. A study in southern Brazil found an even lower prevalence than the present research: 14.8% of women were active before pregnancy, and 12.9% during pregnancy [8]. The prevalence decreased during pregnancy (10.4%, 8.5%, and 6.5% in the first, second, and third trimester), and only 4.3% of participants were active throughout pregnancy. Notably, this study considered only physical exercise and leisure activities, not domestic or work activities [8].

A cohort of 118 pregnant women in Brazil practiced mostly mild-intensity activities at the 16th and at 32nd weeks of pregnancy and all participants were classified as sedentary and performed mostly domestic activities [10]. To assess levels of physical activity, Silva et al. (2007) acculturated and validated the PPAQ with a sample of 305 pregnant Brazilian women and found that 80% performed mild-intensity activities or were sedentary and that mild-intensity activities tended to increase during pregnancy, while moderate activities decreased [9].

The various designs and instruments used and the week or trimester of assessment limit comparisons of studies on the practice of exercise during pregnancy. However, studies investigating the type of exercise have unanimously reported that walking was the most common activity [8,16,28–30]. Walking is an affordable exercise that requires no special equipment or facility. There is no contraindication from its practice during pregnancy, except for the general contraindications for exercise during pregnancy [6]. Several clinical trials have used walking as an intervention to assess the effects of exercise on maternal and perinatal outcomes and have found it to be an effective intervention [31–34].

Water aerobics was the second most frequent type of activity. There seems to be a consensus among pregnant women that water exercise water is beneficial for them and their children [30,35,36]. Especially in Scandinavian countries, swimming is a popular exercise among pregnant women [16,23].

In addition to stretching, which is usually associated with the practice of other activities, women in Brazil reported engaging in Pilates. Likely, women who practiced it before becoming pregnant continued during pregnancy [37]. Pilates is an exercise technique which focuses on body–mind balance, muscle strengthening, flexibility, muscle control, posture, breathing, and the power center of stabilization [37]. Although these practices could have interesting intersections with pregnancy musculoskeletal adaptations, there is insufficient data in the literature to assess the effect on pregnant women.

The large number of women who discontinued the practice of exercise due to pregnancy warrants attention. Although participants were not asked their reason for stopping exercise, possible explanations include medical recommendation to perform exercise only after 12 weeks gestation; discomforts, such as drowsiness, nausea, and fatigue; and fear of miscarriage, which is most common in the first trimester [12,14]. The decrease in physical activity observed in the third trimester might be related to physical changes in the uterus and to fetal growth, resulting in increased body weight and discomforts, such as low back pain, fatigue, and insomnia [13,38].

As observed in this research, the reduction in physical activity level occurred not only in the level of exercise but also in daily activities, such as housework, childcare, transportation, and occupational activities. Different authors have reported that women tend to replace moderate-intensity activities with light-intensity or sedentary activities during pregnancy [10,19]. A systematic review of articles published from 1986 to 2009 identified factors associated with greater participation in exercise during pregnancy: higher education and income level, no other children in the home, white, and activity before pregnancy. Notably, no articles reported on pregnant Brazilian women, although the present results are similar [15]. In this study, planning of pregnancy, prenatal care in private service, and high socioeconomic status were also positively related to exercise.

Exercise guidance and recommendations given to pregnant women in prenatal care is another found worth highlighting. Women who received some counseling were three times more likely to exercise than those who received no guidance. However, even in a sample of healthy pregnant women, less than half received any guidance in prenatal care. And most women who were advised to interrupted exercise did not present comorbidities such as hypertensive disorders of diabetes (other comorbidities such as premature labor, intrauterine growth restriction or hemorrhage diseases) (data not shown). Physicians mostly gave such advice. During each prenatal care visit, medical professionals must address multiple issues, leading to a low prioritization of exercise and pointing to the lack of a multidisciplinary team in prenatal care.

Alternatives to improve the quality of exercise guidance for pregnant women include the participation of a multidisciplinary team in providing educational strategies, support groups, and education about exercise benefits and safety for both pregnant women and medical professionals involved in prenatal care. For instance, in a clinical trial, the intervention group which participated in a program with exercise orientation during prenatal visits increased energy expenditure in physical exercise (1.4 MET-hrs/week), while the control group saw a decrease (-0.3 MET-hrs/wk) throughout pregnancy [39].

Therefore, women of childbearing age, especially those planning a pregnancy, should be encouraged to adopt a healthy lifestyle that includes exercise before conception. In addition, socioeconomic factors, such as education and parity, were also found to be associated with exercise during pregnancy, which supports the need for public health policies that encourage and promote physical activity.

The abundant literature evaluating the effects of exercise on maternal and perinatal outcomes indicates greater benefits than risks [3,5,40]. Our results showed no difference in relation to maternal weight gain. Exercise was not a controlled variable, so its association could not be tested. Weight gain in pregnancy is multifactorial, and the results in relation to exercise effects are still contradictory [41–43].

The C-section rate (58.9%) was higher than rate recommended by the World Health Organization (15%) [44] and supported by recent studies [45]. This issue involves many socio-cultural and economic factors, and although some studies show an association between exercise and a lower rate of surgical delivery [46,47], analysis of the C-section rate and its association with exercise training is limited in this study. In other perinatal outcomes (gestational age, prematurity, newborn weight), there were no differences between pregnant women considered active and sedentary, confirming previous studies’ results that exercise is safe for the mother and the fetus [3,5]. However, the cross-sectional study has limited ability to assess causal effects. In addition, we chose to analyze perinatal variable based on our own exercise criteria which classified women as active if they reported performing exercise regularly (twice or more per week, at least 30 minutes per session) at least in one trimester, instead to use ACOG criteria (150 minutes/week) which might lead to different results.

Although the study design was cross-sectional, women were asked about activities related to pregnancy performed in the last 12 months and the previous 3 months, which could lead to recall bias. However, pregnancy is perceived as a milestone in women’s lives, so they are likely to more easily remember their activities during this period, especially in the interviews conducted in the immediate postpartum period, when information about pregnancy are still relevant to women.

The self-reported nature of the data and the fact of our main outcome (physical exercise pattern) comes from non-validated questionnaire are also limitations that should be acknowledged to interpreter our results. The available validated questionnaires comprise different aspects of physical activity and a limited period of time, such the last 7 days, on e or three months [20–22] which is insufficient to embrace the whole gestation, leading the need of longitudinal studies.

Although future longitudinal studies are needed to address these limitations, this large study highlights the need to urge reproductive-age women to engage in a healthy, active lifestyle before pregnancy and to use prenatal visits as important opportunities to encourage these new healthy habits.

## Supporting Information

S1 File Questionnaire of evaluation of physical exercise during pregnancy. (PDF)
