# Correction: Physical Activity Patterns and Factors Related to Exercise during Pregnancy: A Cross Sectional Study

**DOI:** 10.1371/journal.pone.0133564

**Published:** 2015-07-15

**Authors:** Simony Lira Nascimento, Fernanda Garanhani Surita, Ana Carolina Godoy, Karina Tamy Kasawara, Sirlei Siani Morais

There are errors in the Funding section. The correct funding information is as follows: The publication costs were supported by Fundacão de Amparo à Pesquisa do Estado de São Paulo (FAPESP), process number: 2015/09800-5. The funders had no role in study design, data collection, analysis, decision to publish, or preparation of the manuscript.
