# Supplementary material for: Physical Activity Patterns and Factors Related to Exercise during Pregnancy: A Cross Sectional Study
Source: PLoS One. 2015 Jun 17;10(6):e0128953. doi: 10.1371/journal.pone.0128953 (PMC4470997; doi:10.1371/journal.pone.0128953)
Supplement: S1 File — (PDF) [file pone.0128953.s001.pdf]

Date: □□□□/□□□/□□ □□□□□□

EX&PREG

EVALUATION OF PHYSICAL EXERCISE DURING PREGNANCY

1. In the three months before you were pregnant did you exercise regularly (2-3 times per week for at least 3 consecutive months)?

[1] Yes [2] No What type of exercise?: ____ **(if <u>No</u>, skip to question 3)**

2. How did you behave in relation to the exercises after finding out you were pregnant?

[1] I continued doing exercises [2] I stopped the exercises [3] I continued to exercise, but slowed down

3. During prenatal visits, did you receive advice about physical exercise?

[1] Yes [2] No

4. What healthcare provider(s) gave you advice about exercise?

[1] Doctor [2] Nurse/midwife [3] Physiotherapist [4] Gym or personal trainer [5] Other (describe) ______________________

5. How was this guidance offered?

[1] Individual conversation [2] Conversation in a group with other pregnant women [3] Brochure [4] Videos [5] Other (describe) ______________________

6. At any time during this pregnancy did you practice some kind of exercise such as walking, aerobics, stretching, or prenatal exercise class?

[1] Yes [2] No **(if you did not exercise, skip to question 12.)**

	A) Which of the activities listed below did you do in your first trimester? (Check one or more options)	B) During this period, how many days did you exercise per week??	C) How many minutes per day did you exercise?	D) How strenuous do you think your exercising was? Rate each one from 1 to 10 according to the Borg scale at the bottom of the questionnaire.
7. Did you practice exercise regularly from the 1st to 3rd month of pregnancy (first trimester)?	[1] Walking	_____ days/ week	_____ minutes/ day	[1] [2] [3] [4] [5] [6] [7] [8] [9] [10]
[1] yes [2] No	[2] Running	_____ days/ week	_____ minutes/ day	[1] [2] [3] [4] [5] [6] [7] [8] [9] [10]
(if you did not exercise in the first trimester skip to question **5**)	[3] Swimming	_____ days/ week	_____ minutes/ day	[1] [2] [3] [4] [5] [6] [7] [8] [9] [10]
	[4] Water aerobics	_____ days/ week	_____ minutes/ day	[1] [2] [3] [4] [5] [6] [7] [8] [9] [10]
	[5] Stretching	_____ days/ week	_____ minutes/ day	[1] [2] [3] [4] [5] [6] [7] [8] [9] [10]
	[6] Weightlifting	_____ days/ week	_____ minutes/ day	[1] [2] [3] [4] [5] [6] [7] [8] [9] [10]
	[7] Cycling	_____ days/ week	_____ minutes/ day	[1] [2] [3] [4] [5] [6] [7] [8] [9] [10]
	[8] prenatal exercise class	_____ days/ week	_____ minutes/ day	[1] [2] [3] [4] [5] [6] [7] [8] [9] [10]
	[9] Pelvic floor exercises	_____ days/ week	_____ minutes/ day	[1] [2] [3] [4] [5] [6] [7] [8] [9] [10]
	[10] Other (describe)________________	_____ days/ week	_____ minutes/ day	[1] [2] [3] [4] [5] [6] [7] [8] [9] [10]

Date: □□□□/□□□/□□ □□□□□□

8. Did you practice exercises regularly from 4th to 6th month of pregnancy (second trimester)?	[1] Walking	_____ days/ week	_____ minutes/ day	[1] [2] [3] [4] [5] [6] [7] [8] [9] [10]
[1] yes [2] No	[2] Running	_____ days/ week	_____ minutes/ day	[1] [2] [3] [4] [5] [6] [7] [8] [9] [10]
(if you did not exercise in the second trimester skip to question **6**)	[3] Swimming	_____ days/ week	_____ minutes/ day	[1] [2] [3] [4] [5] [6] [7] [8] [9] [10]
	[4] Water aerobics	_____ days/ week	_____ minutes/ day	[1] [2] [3] [4] [5] [6] [7] [8] [9] [10]
	[5] Stretching	_____ days/ week	_____ minutes/ day	[1] [2] [3] [4] [5] [6] [7] [8] [9] [10]
	[6] Weightlifting	_____ days/ week	_____ minutes/ day	[1] [2] [3] [4] [5] [6] [7] [8] [9] [10]
	[7] Cycling	_____ days/ week	_____ minutes/ day	[1] [2] [3] [4] [5] [6] [7] [8] [9] [10]
	[8] prenatal exercise class	_____ days/ week	_____ minutes/ day	[1] [2] [3] [4] [5] [6] [7] [8] [9] [10]
	[9] Pelvic floor exercises	_____ days/ week	_____ minutes/ day	[1] [2] [3] [4] [5] [6] [7] [8] [9] [10]
	[10] Other (describe)________________	_____ days/ week	_____ minutes/ day	[1] [2] [3] [4] [5] [6] [7] [8] [9] [10]
9. Did you practice exercises regularly from 7th to 9th month of pregnancy (third trimester)?	[1] Walking	_____ days/ week	_____ minutes/ day	[1] [2] [3] [4] [5] [6] [7] [8] [9] [10]
[1] yes [2] No	[2] Running	_____ days/ week	_____ minutes/ day	[1] [2] [3] [4] [5] [6] [7] [8] [9] [10]
(if you did not exercise in the third trimester skip to question **7**)	[3] Swimming	_____ days/ week	_____ minutes/ day	[1] [2] [3] [4] [5] [6] [7] [8] [9] [10]
	[4] Water aerobics	_____ days/ week	_____ minutes/ day	[1] [2] [3] [4] [5] [6] [7] [8] [9] [10]
	[5] Stretching	_____ days/ week	_____ minutes/ day	[1] [2] [3] [4] [5] [6] [7] [8] [9] [10]
	[6] Weightlifting	_____ days/ week	_____ minutes/ day	[1] [2] [3] [4] [5] [6] [7] [8] [9] [10]
	[7] Cycling	_____ days/ week	_____ minutes/ day	[1] [2] [3] [4] [5] [6] [7] [8] [9] [10]
	[8] prenatal exercise class	_____ days/ week	_____ minutes/ day	[1] [2] [3] [4] [5] [6] [7] [8] [9] [10]
	[9] Pelvic floor exercises	_____ days/ week	_____ minutes/ day	[1] [2] [3] [4] [5] [6] [7] [8] [9] [10]
	[10] Other (describe)________________	_____ days/ week	_____ minutes/ day	[1] [2] [3] [4] [5] [6] [7] [8] [9] [10]

10. During prenatal visits, did you receive advice to stop exercising? [1] Yes [2] No

11. During prenatal visits, were you directed to slow down or decrease the intensity of your exercising? [1] Yes [2] No

12. Did someone help you to complete this questionnaire? [1] Yes [2] No Who?______________________

Thank you for participating in this study. Your contribution is greatly appreciated!
